# Dr. Langslow, on Apoplexy, in Reply

**Published:** 1802-05

**Authors:** R. Langslow

**Affiliations:** Halesworth


GENTLEMEN,

OWING to the neglect of my Bookſeller, I did not receive the laſt Number of your valuable Work till the 7th inſtant; this circumſtance, united to my profeſſional avocations, precludes me from anſwering at preſent, ſo fully as I could wiſh, the *eccentric* though elaborate Communication of your Correſpondent Pyrrho. I call it eccentric, becauſe I think he is himſelf ſceptically inclined to the doctrines he has there advanced; for, ſurely, a more wild and fanciful hypotheſis than the idea of the Cauſe of Apoplexy originating in the Lungs, from defective oxygenation* of the ſyſtem, was never before *ſeriouſly* promulgated. Some of my medical neighbours, judging I ſuppoſe from the peculiarity of diction, together with the marked predilection evinced by the author for *antique* authorities, attribute it to the pen of Mr. Crowfoot's patron, Dr. G. Be this however as it may, I ſhall be allowed to aſk him, why any Controvertiſt, upon ſo laudable and intereſting a ſubject as that of Apoplexy, ſhould wiſh to enter the liſts under a maſk? We aſſuredly ought to hope, that every Diſputant who has ſignalized himſelf on this important occaſion, has been actuated by a ſincere deſire of promoting the cauſe of truth, ſcience, and humanity; and ſuch being his motives, he need not feel himſelf diſhonoured by the publicity of his name, notwithſtanding his well-meant exertions might ultimately fail of ſucceſs; and we naturally infer, that when a Diſputant, like this author, advances opinions which he is unwilling to ſanction with his *real* ſignature, that he is imitating, if not ſurpaſſing, his prototype in Pyrrhoniſm, by not only diſbelieving the doctrines of his cotemporaries, but alſo entertaining doubts† of the validity

---

* Do not the countenances of thoſe perſons ſuppoſed to be prediſpoſed to Apoplexy, indicate exceſſive rather than defective oxygenation?

† At page 336, Pyrrho admits his Communication to be a Table of Doubts!

lidity of thofe opinions he has himfelf attempted to promulgate. Pyrrho faid, "Examine all things, determine nothing." But hear what St. Paul fays: "Prove all things, and hold faft to that which is right." How noble, juft, and exemplary are the fentiments of the Chriftian Apoftle, when compared with thofe of the fceptical Greek philofopher.

So much has been urged in the courfe of this difpute about perfonal rancour and contemptuous language, that I feel myfelf obliged, once more, to repeat my affertion in the moft pofitive terms, that when I forbid the ufe of the emetic in the Cafe in queftion, I was actuated *solely* by an anxious wifh to preferve a truly valuable life. Nay, could it be otherwife? At the moment the order iffued from my lips, Mr. C's perfon was unknown to me, as well as the fact of his having *advised* the Emetic;* and my motives for perfevering in the Controverfy are affuredly thofe of benevolence and humanity towards mankind, as I confider the extrication of my own character from the unmerited attack it has experienced only in a fecondary point of view; and if I ultimately fucceed in abolifhing what I truft is by this time generally confidered as a dangerous and injudicious practice, namely, the adminiftration of Vomits in Apoplexy, I fhall be much gratified, and feel myfelf amply compenfated for my trouble and exertions, in the reflection of having deferved well of my country, as well as confolation for the unmerited obloquy and odium my uncandid adverfaries have laboured fo induftrioufly to load me with. I may probably be allowed in this place to introduce a Cafe ftill farther illuftrative of my opinion, felected from others intended for publication in my next work, the particulars of which I have been favoured with by a truly intelligent, learned, and refpectable Divine, the friend and acquaintance of the noble family where it occurred.

The father of a much and juftly refpected Nobleman in this neighbourhood, was attacked with real Apoplexy in his own park; the family furgeon, refiding only a mile diftant, was called to his relief; he, with great propriety, bled him plentifully upon the fpot where it occurred, the effect of which was a fpeedy alleviation of the moft alarming fymptoms; he was then cautioufly and gently removed to the houfe, and farther medical aid required; the refult of which was the immediate adminiftration of a vomit, moft probably to remove the naufea and ficknefs which always fupervene in cafes where the brain fuftains compreffion to a *certain* degree; but, alas! notwithftanding

* Vide pages 5—15 of the Hiftorical Sketch of the Controverfy, with Notes and Comments, critical and explanatory, fold by Cadell and Davies London, price 1s. 6d.

ſtanding, previouſly to the exhibition of the emetic, hopes of recovery were entertained, the vomit no ſooner began to operate than a return and increaſe of the unfavourable appearances took place, and the patient ſpeedily expired. The Apothecary, with that truly laudable liberality and candour that will ever reflect ſplendor on his name, has repeatedly aſſured the reſpectable Divine above alluded to, that he had always regretted the having had recourſe to the exhibition of the emetic on that occaſion; for, however well intended, he now entertained no doubts of its having expedited the fatal event, if not abſolutely occaſioned it. Now, was every medical gentleman actuated by ſimilar motives upon ſuch occaſions, and to acknowledge their erroneous practice with the ſame ingenuous candour as this honeſt and worthy Apothecary has done, the progreſs of improvement in the Medical Art would be much more rapid even than what the preſent enlightened period can boaſt of, and our arrival at the acme of perfection in it be greatly and deſirably expedited.

From the preſſure of profeſſional engagements, I muſt, for the preſent, beg leave principally to refer your numerous readers to my Hiſtorical Sketch of this important Controverſy, ſold by Cadell and Davis, London; as, in the Notes and Comments of that Work, I am inclined to think they will find many parts of the ſingular doctrines of Pyrrho already, in ſome meaſure, combated; and in a work I ſhall ſhortly have the honour of iſſuing from the Preſs, I ſhall ſtill farther inveſtigate their merits and foundation; and though, in ſuch Inquiry, I may not, like Pyrrho, be able to boaſt of a very intimate acquaintance with the doctrines and opinions of a Valiſnerius, a Helmont, a Riverius, a Caldani, or a Lupi, &c. upon Apoplexy, I ſhall however, I truſt, readily make it appear, that in my *mode of treating* this dangerous malady, I have the ſanction and ſupport of a Hoffman, a Cullen, a Fordyce, and a Portal; and let me aſk Pyrrho, whether the latter authorities, aided and enlightened as they have been by the anatomical, phyſiological, and pathological diſcoveries of late years, are not of more weight and conſequence than the opinions of moſt of thoſe writers, (unaided by ſuch diſcoveries) which with ſuch an affectation of antique erudition he has taken ſuch unuſual pains to ſelect? though I have my doubts whether Pyrrho himſelf can convince us, that either of his admired authors ever attempted to transfer the ſource of Apoplexy from the brain to the lungs; and I cannot repreſs my applauſe of the Editors for their expreſſing their wiſhes for an opportunity of preſenting *such a Case* to their Readers.

Notwith-

Notwithſtanding the hurry in which I am compelled to write, I cannot take leave of Pyrrho without referring him to the Notes introduced in the Hiſtorical Sketch for proofs of that forbearance, candour, and liberality he ſo vauntingly boaſts of in the conduct of his friend, Mr. Crowfoot. The truth of the remarks adduced in my Publication, as well as thoſe contained in my Letter to Dr. Girdleſtone and Mr. C. publiſhed in the Ipſwich Journal, of the 2d of January laſt, in anſwer to their Advertiſement of the 26th of December, 1801, and ſince copied into the Hiſtorical Sketch, p. 44 to 48 of that Work, and their ſtill remaining unrefuted and uncontradicted, are ſurely unqueſtionable proofs of their foundation in truth. If Mr. Crowfoot was originally juſtified in refuſing an interview with a Phyſician he never ſpoke to, and probably had never ſeen, why did he ſubſequently court *such* interviews *unnecessarily*, till his farther attendance upon the patient was abſolutely forbidden by her huſband, without my knowledge or approbation?

At page 330, I am accuſed of ſpeaking vauntingly and contemptuouſly of my Opponents; I do not plead guilty to the charge, as I am unconſcious of having done ſo, and aſſuredly did not intend it; though, indeed, had ſuch been the fact, ſome allowance ought to have been made for the feelings of a Phyſician, attacked and traduced as I was, without the *slightest cause*, by an ex parte Statement circulated clandeſtinely among the friends of Mr. C. and which has been *proved*, in my Anſwer, to have been compoſed principally of falſhoods and miſrepreſentations: Under ſuch treatment, would it have been wonderful if I had been betrayed into an undue degree of warmth and acrimony? though, upon a calm retroſpective view of my writings, I cannot diſcover any good ground for ſuch cenſure. However, I am ready to ſubmit my conduct upon this occaſion to that Tribunal which rarely errs; * and if the Public pronounce me guilty, I ſhall readily and cheerfully make every reparation for the injury ſuſtained, which can reaſonably and honourably be required of me. I have, therefore, collected all the facts in my Hiſtorical Sketch, and thus the merits of the reſpective parties engaged in the Controverſy are now placed ſub judice. I ſhall wait the deciſion with patience, and ſubmit to the Decree, even ſhould it unexpectedly be pronounced againſt me, with reſignation and without complaint.

For

* Who does Ariſtarchus, jun. allude to in his well-timed cenſure upon illiberal and acrimonious language?

For the refutation of thofe arguments adduced by P. againft the idea of emetics increafing the determination to the head, and thereby tending to increafe exudation from the exhalants, or effufion from vafcular rupture, I fhall beg leave for the prefent to refer him to the ingenious Obfervations of the intelligent Dr. Mofsman, in No. 37 of your Journal; nay, their tendency to impede the return of blood from the head, *admitted by P. himself*, is an effect fufficient to deter us from their *ufe* in Apoplexy, even did they not impel the blood with violence into the head during the action of vomiting, though I am of opinion P. has convinced but few of his readers that they do *not* poffefs the *latter power* and tendency.

At page 333, P. feems to ridicule the idea of my attaching the leaft importance to the diftinction between fanguineous and ferous apoplexy, becaufe the abftraction of blood is recommended in *both* cafes; but it furely does not follow, that the evacuation may be carried with equal propriety to the fame extent in *both* affections. That the fame kind of remedy is indicated I will allow, because the caufe of the difeafe is the fame in both instances, *preffure on the brain*; but furely the abftraction of fix or twenty ounces of blood from the fyftem, will produce ultimate effects *widely* different; the latter quantity may be expedient and proper in fanguineous Apoplexy produced by effufion from vafcular rupture, in a full fanguine plethoric conftitution, but in a cafe of ferous Apoplexy induced in a different habit, a fimilar abftraction of blood might induce a fatal torpor—The propriety and importance therefore of diftinguifhing ferous from fanguineous Apoplexy, will, I truft, no longer be difputed or ridiculed by Pyrrho; he cannot from the writings of Morgagni or Portal make it appear, that though they very judicioufly recommend bleeding in *both cafes*, that they confider it as advifeable in the *same* proportion. At page 146, vol. iii. of Cullen's Practice, Pyrrho will difcover that enlightened author ftating as follows: "From the view I have now given of the caufes of Apoplexy, it will readily appear that *there is a foundation* for the common diftinction of this difeafe into the two kinds of fanguine and ferous." This being the opinion of the immortal Cullen, and my having already proved the neceffity of apportioning the abftraction of blood to the nature of the compreffing caufe, it will be clearly evinced that the ridicule attempted to be attached to the importance of this diftinction is *proved* to be ill timed and unfounded.

In anfwer to the obfervation of P. at page 334, objecting to the probability of over diftenfion *ever* proving a fource of extravafation, that opinion, *if juft*, operates more powerfully againft the fupporters of the doctrine of congeftion, turgefcence, and expanſion of the veſſels of the brain being the ſource of compreſſion on that organ, in Apoplexy, than it does againſt my opinion that the moſt frequent cauſe of Apoplexy, when the diſeaſe does not terminate in death, or paralyſis, is exudation from the orifices of the exhaling arteries.

At page 335, Pyrrho expreſſes his doubts * of an abſorbing ſyſtem exiſting in the brain, becauſe lymphatics have never been there demonſtrated either by diſſection or injection; if P. will trouble himſelf to refer to a note at page 28 of my Hiſtorical Sketch, he will there find it ſtated, that notwithſtanding the lymphatics of that viſcus have hitherto eluded the knife and the ſyringe of the Anatomiſt, it is poſſible this may have ariſen from their extreme minuteneſs in that organ, as from analogy we cannot eaſily relinquiſh the idea of their exiſtence; but admitting, for the ſake of argument, that they do not exiſt, is it not poſſible the inhaling viſcus may perform this office in that particular organ? The beſt Phyſiologiſts have entertained this opinion; and at page 144 of vol. iii. Cullen has admitted the probability of the fact; and for my mode of accounting for the ſudden and in ſome caſes ſpeedy reſtoration of a *portion* of the ſenſorial functions in Apoplexy, particularly that of *ſpeech*, I muſt refer you to my reply to Mr. Atkinſon, which appeared in the laſt Number (37) of your Journal.

I ſhall next advert to P's comparative view of the effects of bleeding in the different caſes of Apoplexy he has met with, introduced page 336, where he again expreſſes his *doubts* whether thoſe which have loſt the greateſt quantity of blood, have not proved the moſt fatal. I have no objection to concede to P. the correctneſs of this calculation, as it is reaſonable to infer that a judicious practitioner would abſtract the greateſt proportion of blood, in caſes of ſanguineous Apoplexy, and I have formerly aſſerted that I believe few *such* caſes terminate favourably.

I cannot, however, drop my pen, without complimenting the author upon his ſuddenly aſſumed candor and liberality, where at 337, he expreſſes his gratification that his communication may tend to repreſs in tbe *mover* of this Controverſy, boldneſs of aſſertion, and acrimony of invective: That his friend Mr. C. muſt be the perſon alluded to, has been repeatedly *proved* and aſſerted without refutation or contradiction; Mr. C. had aſſuredly recourſe *firſt* to the pen, the news-paper, and the preſs, and is therefore *entitled* to thoſe applauſes and commendations due

* Let it be recollected that the whole of his communication is ſceptic-like, a ſeries or collection of Doubts!

due to the original promoter of the prefent Inquiry into the caufes and treatment of Apoplexy. I am, however, at a lofs to difcover in what part of my publication on this fubject, I have afferted that Apoplexy is to be removed or cured with Mefmerian, or Perkinean facility; P. will probably, in fome future communication, have the goodnefs to point out the page. I have uniformly lamented the fatality and danger generally attendant upon this much and juftly dreaded malady, though I can by no means accord with P. in his *doubts* of the frequent, though perhaps not uniform, good effects of general and local bleedings, blifters, laxatives, and cathartic injections in *Apoplexy.*

I fhall now trouble you with a few lines, in reply to Mr. Crowfoot's laft paper, and then take my leave of the fubject for the prefent month. Mr. C. commences with a complaint againft that ingenious and fcientific phyfician Dr. Moffman, who will doubtlefs, in fome future Number, with his accuftomed ability and candor, repel the unmerited attack, if he conceives it worthy his notice. He then proceeds to ftate his anxiety to relinquifh the Controverfy; his beft friends think it would have been to his advantage had he never began it. His oftenfible motive however for fo doing is ftill more extraordinary if not myfterious, for he ftates, "that Controverfy is *impracticable*, and Refutation *ufelefs*," page 361, No. 38. Now, on the contrary, that Controverfy has been, and is ftill *practicable*, has been completely evinced during the laft five months by our having unremittingly perfevered in it; and that the refutation of erroneous opinions in the mode of treating fo dangerous a malady as that of Apoplexy will be ufeful, I truft no man, Mr. C. excepted, will attempt to deny. Had Mr. C. reverfed his obfervation and faid, that farther controverfy upon this occafion is now *ufelefs*, as proof of the utility of Vomits in Apoplexy is *impracticable*, he would have been nearer the truth, and affuredly have been more readily underftood. Mr. C. next complains of my intemperate and contemptuous language towards himfelf, Meff. Wilkinfon and Atkinfon, whom, it feems, he wifhes to confider as converts to his opinions. Had my reply to Mr. Wilkinfon impreffed him with a fimilar idea, he would in all probability have made an appropriate rejoinder. That Mr. A. did not think my language intemperate or contemptuous, is evinced by his Anfwer in the laft Number, where, at page 365, he obferves, Dr. L's defence againft Mr. C. appeared *to him*, with fome exceptions, to be pregnant with utility and juft difcrimination; and to prove that Mr. C. has deceived himfelf in fuppofing Mr. A. a convert to his opinions, allow me to refer him to his obfervations on what he calls the *extraordi-*

*nary doctrines* of Mr. Crowfoot, at page 365 of the fame Number, and where he afferts, that he conceives an emetic in Apoplexy a moft objectionable and injudicious remedy; "and if Mr. C. contends that there is no *preffure upon the brain*, he cannot imagine the cafe either nervous, fanguineous, or ferous Apoplexy, for that, certainly no cafe of *real* Apoplexy *ever occurred* but was caufed by an affection of the brain." I truft thefe rational, and intelligent obfervations of Mr. A. do not indicate that he conceived my language either intemperate or contemptuous, or that he is a Convert to the opinions of Mr. C.

Mr. C. next proceeds to point out, what he is pleafed to term contradictions* in my publications; and charges me with ftating, that in my *explanation* I afferted that the two words, extravafation and blood, were not made ufe of by me, in the prefence of the family, the day I was called to the lady in queftion, becaufe the parties prefent did not hear me; and it is from this miftaken idea that he concludes I had at one time changed my ground, and abandoned the opinion of extravafation being the moft general caufe of real Apoplexy, as he declares I afterwards ftate, that "the expreffion is directly repugnant to my opinion." Now, had I really written and made the obfervation Mr. C. charges me with, his inference would be juft, and his triumph complete; but unfortunately for the comprehenfion or veracity of Mr. C. I fhall prefently prove that his premifes were falfe, and his inference confequently erroneous. If Mr. C. will trouble himfelf to refer to my explanatory Letter, a copy of which is inferted at page 37 of the Hiftorical Sketch, he will find the words ufed by me were, *not extravasation* and blood, but *confiderable and blood*; I never denied the *extravasation*, only that of its being *confiderable*. I always admitted, and ever confidered, the caufe of the affection to have been "preffure upon the brain," induced by *exuded*, confequently *extravafated* ferum, as the term extravafation applies with equal propriety whether the compreffing fluid be blood or ferum; and I confider myfelf juftified in denying Mr. C's firft ftatement to *be true*, where he afferts that I faid there was *confiderable* extravafation of *blood* upon the brain, becaufe the affertion would have been repugnant to my opinion; for though I believed in the *extravasation*, I never confidered it as *confiderable*, or that it was *blood*; though, had I feen the patient when the attack firft took place, my opinion might have been different. Before fhe was bled, the

* Would not Mr. C. be more ufefully engaged in endeavouring to refute my arguments than in fearching for typographical errors, or fuch as may be the refult of profeffional hurry?

the ſymptoms were ſo alarming and violent, that a practitioner might have been juſtified in ſuppoſing the extravaſation conſiderable, and to have been induced by vaſcular rupture; but at the period of my arrival, near two hours after ſhe was bled, the alarming ſymptoms were ſo much alleviated, and the appearances of compreſſion on the brain ſo much diminiſhed, that I was induced to conclude, from the ſpeedy abſorption of a *portion* of the *extravasated* fluid, that its nature muſt have been ſerous, and the quantity by no means large. By a reference to the explanatory Letter alluded to, my opinion and meaning may be ſtill more clearly underſtood; and if Mr. C. really conſidered my Statement as denying the exiſtence of *extravasation* in the caſe in queſtion, we muſt ever lament his defective comprehenſion and diſcrimination. I truſt, however, a ſimilar conſtruction to that of Mr. C's, has by no other Readers been applied to the language of my Explanatory Letter. What I have already written will ſufficiently explain and elucidate the ſucceeding Remarks of Mr. C. till we arrive at the ſentence, page 362, where Mr. C. ſays, if the effuſion had not at firſt been aſſerted to be *considerable*, how are we to reconcile the principal part being abſorbed, and *now* but little remaining? Is it ſo difficult to comprehend how the principal part even of an *inconsiderable* quantity of extravaſated fluid may be abſorbed, and yet admit of a *little* to remain? as very *small* quantities of a fluid will allow of a diviſion or ſeparation of parts.

Mr. C. then ſays, it is difficult to imagine how that could be denied, which had not been affirmed. I again call upon Mr. C. to point out where, and when, I *denied the extravasation* of ſerum in the caſe in queſtion. Mr. C. ſoon after ſtates, that there was never any reaſon to doubt the correctneſs of the opinion formed by Mr. Primroſe in the caſe of this lady. Now it is probably in the recollection of moſt of your Readers, that Mr. Primroſe was the firſt who conſidered and determined the caſe of our patient to be *real* Apoplexy; Mr. C. confirmed his opinion when he arrived, and I agreed in opinion with both upon *this point*. Now, upon referring to Mr. C's ſtatement of the caſe, copied into my Hiſtorical Sketch, p. 2 to 13, the Reader will find, in a Note inſerted page 3 of the Sketch, that he, Mr. C. declares he *admits* the caſe to be Apoplexy, *to avoid cavilling*; but *now* aſſerts and ſupports the accuracy and correctneſs of the ſame gentleman he had ſo aſperſed, by declaring that he originally *admitted* the caſe to be Apoplexy, *to avoid cavilling*.* In the ſucceeding ſentence, Mr. C. is pleaſed to

* Vide my Notes on this conduct of Mr. C. p. 3, 9, and alſo at p. 24 of the Hiſtorical Sketch.

to term my addrefs to Mr. Atkinfon a Phyfical Romance; probably he found it a much more eafy tafk *to attach that title* to it, than to *refute* the arguments* it contained. Mr. Atkinfon feems to entertain a different opinion, and will probably transfer the correction of my numerous errors, and the refutation of my unfounded affertions, to the pen of Mr. C. I truft he will not be offended with me in propofing to apply the concluding paragraph of his letter to his own conduct and behaviour. He ftates, that it is the production of a much *admired* author; I do not recollect it in the works of Addifon, Junius, or any of the celebrated writers I have myfelf perufed; but was I to judge from the expreffion, *impartial reasonings*, I fhould be inclined to attach the credit of the paragraph to Tom Paine; and if my conjecture is correct, and Mr. C. is a real *admirer* of him or his *writings*, I envy him not his literary tafte.

*Halesworth*, *April* 10, 1802. R. LANGSLOW, M. D.

---

### *To the Editors of the Medical and Phyfical Journal.*

* It is rather fingular that both Mr. C. and his friend Dr. G. have applied themfelves more diligently to the difcovery of typographical errors, or contradictions, as they term them, than to the refutation of argument.

