# Errata

**Published:** 1802-07

**Authors:** 


### ERRATA.

Vol. VII. Page 450, line 17, *for* viſcus *read* veins.

